# Weight Gain Is Associated with Medial Contact Site of Subthalamic Stimulation in Parkinson's Disease

**DOI:** 10.1371/journal.pone.0038020

**Published:** 2012-05-30

**Authors:** Filip Růžička, Robert Jech, Lucie Nováková, Dušan Urgošík, Josef Vymazal, Evžen Růžička

**Affiliations:** 1 Department of Neurology and Centre of Clinical Neuroscience, First Faculty of Medicine and General University Hospital, Charles University, Prague, Czech Republic; 2 Department of Stereotactic and Radiation Neurosurgery, Na Homolce Hospital, Prague, Czech Republic; 3 Department of Radiology, Na Homolce Hospital, Prague, Czech Republic; Banner Alzheimer's Institute, United States of America

## Abstract

The aim of our study was to assess changes in body-weight in relation to active electrode contact position in the subthalamic nucleus. Regular body weight measurements were done in 20 patients with advanced Parkinson's disease within a period of 18 months after implantation. T1-weighted (1.5T) magnetic resonance images were used to determine electrode position in the subthalamic nucleus and the Unified Parkinson's disease rating scale (UPDRS-III) was used for motor assessment. The distance of the contacts from the wall of the third ventricle in the mediolateral direction inversely correlated with weight gain (r = −0.55, p<0.01) and with neurostimulation-related motor condition expressed as the contralateral hemi-body UPDRS-III (r = −0.42, p<0.01). Patients with at least one contact within 9.3 mm of the wall experienced significantly greater weight gain (9.4±(SD)4.4 kg, N = 11) than those with both contacts located laterally (3.9±2.7 kg, N = 9) (p<0.001). The position of the active contact is critical not only for motor outcome but is also associated with weight gain, suggesting a regional effect of subthalamic stimulation on adjacent structures involved in the central regulation of energy balance, food intake or reward.

## Introduction

Deep brain stimulation of the subthalamic nucleus (STN-DBS) is a remarkably effective method for treating motor manifestations of advanced Parkinson's disease (PD). In addition, a variety of non-motor effects related to STN-DBS have been described, including weight gain. Although the precise mechanism underlying changes in body weight has yet to be determined, several hypotheses have been advanced [1]. Some authors have suggested that weight gain may be related to changes in medication, especially to the reduction of dopaminergic drugs [2], [3], [4]. Others have emphasized that weight gain may be related to the normalization of energy expenditure due to decreased rigidity and the amelioration of dyskinesia [5], [6]. Additionally, changes in weight could reflect the direct influence of STN-DBS on adjacent structures involved in the regulation of eating behavior or energy balance [3], [5], [7].

It has been proposed that DBS may cause the excitation of axons surrounding the electrode and increased output from stimulated nuclei [8], [9]. The spread of current has been estimated to occupy approximately a 2–4 mm radius around the electrode contact [10], [11], [12]. Given structural and functional complexity of the subthalamic area, it is believed that the diffusion of stimulation current to its different parts plays a role in motor improvement as well as in the various side effects of DBS [10], [11]. From this perspective, it is conceivable that the stimulating DBS electrode could influence body weight, especially if it was close to the structures involved in the regulation of energy expenditure, food intake or reward, such as the lateral hypothalamic area [13], [14], medial forebrain bundle [15] or the limbic part of the STN [16], [17], [18]. Notably, all of these structures lie in the medial part of the subthalamic area [19], [20]. On the other hand, in terms of motor improvement, subthalamic stimulation appears to be most effective in the dorsolateral border of the nucleus (sensorimotor part) [21], [22], [23]. Thus, the position of active contact relative to the intrinsic organization of the STN might differentially contribute to motor effects and weight changes.

Therefore, the aim of our study was to assess whether weight gain observed in PD patients treated by STN-DBS is dependent on the active electrode contact position in the STN, particularly with respect to mediolateral direction.

## Methods

### Patients and weight measurement

Regular body weight measurements were made on the day of surgery and one, two, four, six, twelve and eighteen months after electrode implantation in 20 patients with advanced PD (6 women, 14 men; mean age 56.6±(SD)5.8 years; disease duration 13.2±4.5 years). Demographic data of the patients that participated in the study are summarized in table 1. A maximum change in weight during the study period and weight change at the 18th month were considered in each patient. Weight changes were expressed in absolute values as well as in percentage of initial body weight. Eating related questionnaires were administered at each visit. Food intake, hunger, general appetite and preference for sweet food were rated by patients as (0) without any change, (−1) lower or (+1) higher than at the previous visit. All patients provided written, informed consent for participation in the study and the study was approved by the Ethics Committee of the General University Hospital in Prague, Czech Republic.

**Table 1:** Clinical description of PD patients treated with subthalamic deep brain stimulation.

	gender	age at surgery (yrs)	PD duration before surg. (yrs)	UPDRSIIIs-OFF	UPDRSIIIs-ON	initial BMI (kg/m^2^)	initial body weight (kg)	maximum weight gain (kg)
1	F	53	20	30	17	19,9	53,3	18,3
2	F	63	18	32	17	17,8	50,1	14,9
3	M	65	22	44	24	23,2	84,2	12,3
4	F	61	12	43	28	24,4	65,6	12
5	M	53	15	62	22	22	69,6	9,4
6	F	58	10	37	17	26,6	64,1	7,5
7	M	56	12	41	23	30,9	100	7,2
8	M	57	14	30	26	20,6	71,3	7,2
9	M	55	7	25	18	27,5	86	7
10	M	67	11	32	16	28,2	86,4	6,5
11	F	58	7	37	18	22,4	61,6	6,4
12	F	42	23	53	19	33,3	80	6
13	M	48	14	36	17	21,6	70	5,3
14	M	56	13	39	16	27,7	95,2	4,5
15	M	49	11	25	12	25,1	67,5	4,1
16	M	57	15	44	11	26,8	84,8	3,9
17	M	63	10	35	14	25,8	72,9	3,6
18	M	58	9	36	24	25	76,6	2,1
19	M	57	10	35	12	28,3	86,8	0,2
20	M	55	10	18	5	29,8	112,3	−0,3

F – female, M – male; PD – Parkinson's disease; UPDRS-III – motor subscore of the Unified Parkinson's Disease Rating Scale; sOFF – postoperative off-neurostimulation state; sON – postoperative on-neurostimulation state; BMI – body mass index; initial body weight – body weight assessed before implantation; maximum weight gain – maximum weight change over the whole study period.

### Surgical procedure and stimulation settings

**Figure 1:**
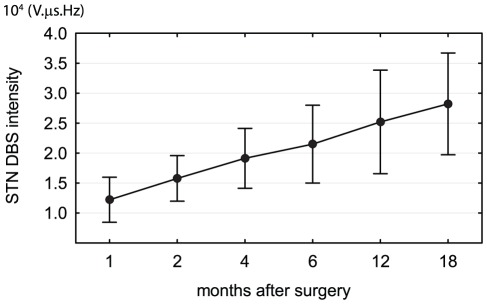
Mean stimulation intensity (±SD) of the STN-DBS at 1, 2, 4, 6, 12 and 18 months after implantation in 20 patients with Parkinson's disease. The stimulation intensity was calculated as the arithmetic product of the I-intensity, u-voltage, d-pulse duration and f-frequency from both hemispheres (uL.dL.fL+uR.dR.fR)/2. The stimulation intensity was gradually increasing during the study to optimize the motor outcome.

Bilateral DBS electrode implantation (model 3389, Medtronic, Minneapolis, MN, USA) was guided by MRI-based stereotaxy, microelectrode recordings and the test stimulation procedure as described elsewhere [24]. Within three days the electrodes were connected to a subcutaneously implanted pulse generator (Kinetra, Medtronic). Stimulation was initiated one month following implantation when each patient underwent standard screening of all electrode contacts in an off-medication state. Finally, one contact on each side and stimulation settings using a monopolar or bipolar (in one patient) setting were selected to obtain the best motor outcome. In the following month, the stimulation intensity was gradually increased (Figure 1) while dopaminergic medication was in most cases reduced to further optimize the motor outcome. For the purpose of our study, stimulation intensity was calculated as the mean of arithmetic products of all the parameters from both neurostimulators (I-intensity, u-voltage, d-pulse duration, f-frequency): I = (u_L_.d_L_.f_L_+u_R_.d_R_.f_R_)/2 [25]. At month 18, the stimulation parameters were 2.8±0.5 V, 60–120 µs and 130 Hz and the mean stimulation intensity was 2.8±0.8. 10^4^ V µs Hz.

### Motor outcome assessment of STN-DBS

Motor status was evaluated using the motor subscore of the Unified Parkinson's Disease Rating Scale (UPDRS-III). Each subject was examined postoperatively under two conditions at least 12 hours after discontinuing all antiparkinsonian drugs: (1) in the off-neurostimulation state (sOFF) and (2) in the on-neurostimulation state (sON). The change of motor status induced by stimulation was expressed as the percentage of UPDRS-III (100-100sON/sOFF). Additionally, hemi-body subscores derived from the UPDRS-III (items 20–26) were calculated as the sum of limb ratings of ridigity, akinesia and tremor, separately for the left and right extremities.

### Assessment of active contact position

Magnetic resonance images were acquired at 1.5 T on a Siemens Avanto system (Siemens, Erlangen, Germany) in each patient approximately one year after DBS implantation. To obtain better image resolution, sagittal (0.9 mm isotropic) and axial (1×1×1.6 mm) T1-weighted magnetization-prepared rapid acquisition gradient-echo (MPRAGE) images were automatically co-registered and averaged using SPM5 software (Wellcome Trust Centre for Neuroimaging, London, UK).

All four contacts (0,1,2,3) of the DBS electrode produced well-defined susceptibility artifacts on the T1-MPRAGE image in each patient [26]. While the coordinates of contacts 0 and 3 were established directly from the center of the distal and proximal artifacts using MRIcro 1.40 software (www.cabiatl.com/mricro), the coordinates of contacts 1 and 2 were calculated. The x-coordinate of each contact was measured from the wall of the third ventricle, whereas the y- and z-coordinates were measured from the midcommisural point. Two coordinate systems, native and normalized, were used in the study. During linear normalization, all dimensions were manually adjusted with respect to the standardized AC-PC length, to the distance of the midcommissural point from the lateral edge of the putamen, and to the distance of the optic tract from the dorsal edge of the putamen. Finally, the active contacts in both hemispheres were plotted on axial (xy), coronal (xz) and sagittal (yz) planes covering the whole subthalamic area.

## Analysis

Statistical analysis was performed using SPSS 14.0.1 software (SPSS Inc, Chicago, IL, USA). For parameters with normal distribution, parametric tests (one sample t-test, paried t-test, Pearson correlation analysis) were used. The others were assessed with the non-parametric tests (Friedman test, Spearman rank correlation analysis).

Primary outcomes of the study were based on the maximum weight gain throughout the study and on the hemibody UPDRS-III in the sON state after initiation of neurostimulation. Their dependence on active contact position was analyzed for each x, y and z-axis separately by Pearson correlation analysis when considering the left and right hemispheres independently, as well as for all active contacts pooled bilaterally taking into account only one active contact (more medial or lateral contact from both hemispheres) in each patient.

In addition, we systematically sought a border dividing the subthalamic area into regions with higher and lower risk of weight gain. To do so, we compared weight gain relative to the active contact position in the subthalamic area divided into two regions of interest (ROI) by a movable yz-plane in the mediolateral direction (x-axis). The iterative general linear model (GLM) was used to compare weight gain in patients with at least one contact within one ROI and patients with both contacts in the other ROI. The factor GENDER and covariates AGE and TIME of postoperative maximum weight gain were included to control for possible confounding effects. The division yz-plane was then successively moved along the x-axis by 0.5–1 mm steps to define a BORDER with lowest p-value. A similar approach was used to compare weight gain considering active contacts in two subthalamic ROIs separated by a movable xz-plane in the anteroposterior direction (y-axis) and by the xy-plane in the ventrodorsal direction (z-axis).

Relationships between body weight, motor performance, eating behavior and intensity of stimulation were assessed separately as secondary outcomes. As they were based on multiple comparisons, the Bonferroni correction was applied whenever appropriate.

## Results

**Figure 2:**
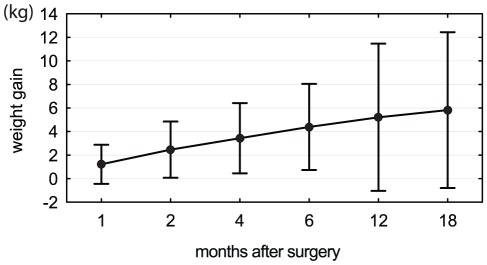
Mean changes in weight after implantation in 20 patients with Parkinson's disease. Body weight gradually increased during the study period. Weight gain represents the difference in weight (±SD) compared to the preoperative state.

After initiation of STN DBS, the UPDRS-III score dropped on average from 36.7±(SD)9.6 (sOFF) to 17.8±5.5 (sON) (T = 7.3, p<10^−7^) showing good efficacy of neurostimulation treatment. The maximum change in body weight in the eighteen-month period after implantation was on average +6.9 kg±4.5 kg (−0.3 to +18.3 kg) and was strongly significant (T = 6.6, p<10^−5^). Despite gradually increasing weight during the entire study period (Figure 2), nine patients reached the maximum body weight within the first 6 months after surgery, five patients in months 6–12 and six patients in months 12–18 after surgery.

**Figure 3:**
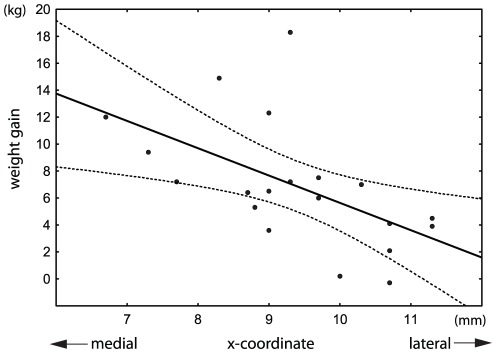
Weight gain in 20 patients with Parkinson's disease in relation to the mediolateral position of the active contact with bilateral STN-DBS (r = −0.55, p<0.01). Only one active contact (more medial contact from both hemispheres) was used in each patient. The x-coordinate represents the distance of the active contact from the wall of the third ventricle. Each millimeter in the medial direction was associated on average with a 1.6-kg increase in body weight. Dotted lines denote the 95% confidence interval of the regression line.

**Figure 4:**
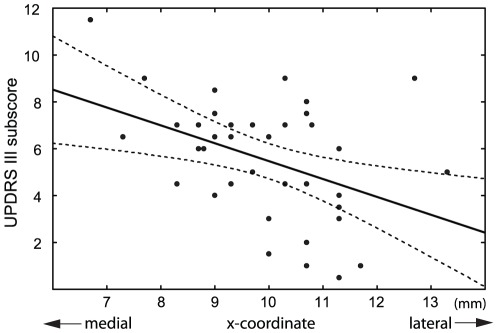
Hemi-body UPDRS-III subscores in the sON condition after overnight withdrawal of dopaminergic therapy in relation to the mediolateral position of the contralateral active contact. After initiation of STN-DBS, the hemi-body side with the lowest motor score (best motor condition) had the contralateral contacts located more laterally from the wall of the third ventricle (r = −0.42, p<0.01). Dotted lines denote the 95% confidence interval of the regression line.

As the analyses of active contact coordinates derived from native and normalized approaches yielded similar results, only statistics based on coordinates in native space are reported. In individual patients, the maximum weight gain correlated inversely along the x-axis with the distance of the active contact from the wall of the third ventricle in the left hemisphere (r = −0.48, p<0.05), right hemisphere (r = −0.50, p<0.05), and in pooled data (r = −0.55, p<0.01) if only more medial active contact regardless to hemisphere was considered (Figure 3). Similar results were obtained for maximum weight gain expressed in percentage of initial body weight as well as when considering weight gain at the end of the 18th month. In addition, the hemi-body UPDRS-III subscores in sON condition inversely correlated with the distance of the contralateral active contact from the wall of the third ventricle in the mediolateral direction (r = −0.42, p<0.01) (Figure 4). However, none of these parameters showed any relation to the active contact position along the y-axis or z-axis.

**Figure 5:**
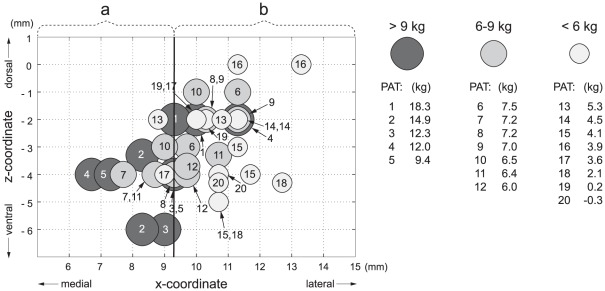
Bilateral STN-DBS active contact positions of 20 patients with Parkinson's disease plotted in the coronal plane with respect to weight gain. Patients (N = 11) with at least one active contact (a) placed within 9.3-mm of the wall of the third ventricle gained significantly more weight than patients (N = 9) with both contacts (b) located more laterally (p<0.001).

With the iterative moving plane approach, we found a border orthogonal to the x-axis dividing the subthalamic area into two ROIs that differed in postoperative weight gain. Patients with at least one active contact within 9.3 mm of the wall of the third ventricle demonstrated significantly greater weight gain (9.4±4.4 kg, N = 11) than those patients with both contacts located more laterally from the wall (3.9±2.7 kg, N = 9) (GLM, factor BORDER: F = 16.1, p<0.001)(Figure 5). The postoperative maximum weight gain significantly differed between genders, with a greater increase in women (N = 6, 10.9±(SD)4.8 kg) than in men (N = 14, 5.2±3.4 kg) (GLM, factor: GENDER, F = 10.7, p<0.01). However, no other covariates (factor AGE: F = 0.001, p = 0.99; factor TIME: F = 0.002, p = 0.96) nor interactions between BORDER, GENDER, AGE and TIME were significant.

In addition, the postoperative maximum weight gain in all patients inversely correlated with preoperative body weight (r = −0.62, p<0.05 corrected). Maximum weight gain did not significantly depend on UPDRS-III improvement after switching the stimulation on (r = −38, p = 0.1), and no correlation between weight gain at the 18th month and stimulation intensity was found. Analysis of eating behavior failed to demonstrate any change in hunger, appetite, preference for sweet food or food intake in our patients. However, there was a positive correlation between food intake and body-weight gain at the 18th month (rho = 0.66, P<0.05 corrected).

## Discussion

We observed weight gain inversely related to the distance of the contacts from the wall of the third ventricle (Figure 3), and patients with at least one contact located medially in the STN experienced significantly greater weight gain than those with both active contacts located laterally (Figure 5). Thus, our results are consistent with the hypothesis that STN-DBS exerts a regional effect on adjacent structures involved in energy balance. In addition, our findings are also in agreement with reports of weight gain observed after unilateral STN-DBS [27], [28]. As the position of each implanted electrode was verified by intraoperative microrecording and DBS caused clear motor improvement, we believe that our observations are not affected by electrode misplacement outside the STN. However, no correlation between stimulation intensity (Figure 1) and weight gain (Figure 2) was found in our study. This may be partly explained by low variability of stimulation parameters between patients or limited size of the patient group.

The maximum weight gain in our study was significantly larger in women than in men. Although women may be more susceptible to weight gain [29], previous studies have proven no significant sex-related differences in weight gain after unilateral or bilateral STN-DBS [2], [4], [5], [7], [27], [28]. These findings are in agreement with our observation that weight gain in all six women of our study was associated with the medial contact site and that no interaction between active contact position and gender was found.

Similar to other studies [21], [22], [23], we found an inverse correlation between unilateral motor outcome (measured for rigidity, akinesia and tremor using hemi-body UPDRS-III subscore) and contralateral position of the active contact (Figure 4). Thus, patients with the lowest motor score (best motor condition) had contacts located more laterally from the wall of the third ventricle. Such results most likely reflect the internal organization of the STN with the sensorimotor part located dorsolaterally in the nucleus [20].

However, we did not observe any significant correlation between weight gain and change in UPDRS-III score. This finding is consistent with those published previously [2], [4], [30] and may indicate that the connection between changes in weight and motor outcomes is not as straightforward as has been proposed [31]. Unrelated weight gain to motor outcome was also shown in another study in which weight gain was more pronounced in patients with subthalamic stimulation than in patients with pallidal stimulation, despite similar motor improvement in both groups [30]. Thus, additional factors likely contribute to greater weight gain in subthalamic stimulation.

The central mechanism by which STN-DBS might cause weight gain remains unclear. It could be hypothesized that the spread of stimulation current beyond the borders of the STN may influence the hypothalamic regulation of energy metabolism or the homeostatic pathway of food intake. However, there are so far only a few studies on the effects of long-term STN-DBS on autonomic [32], [33], [34] or hormonal systems [35], and they have provided no clear explanation for weight gain.

Conversely, increased food intake by non-homeostatic or reward mechanisms may also provide a compelling hypothesis. The medial tip of the STN is involved in basal ganglia limbic and motivational functions [16], [17], [18], [36]. It is connected to key structures of the reward system such as the ventral pallidum and the ventral tegmental area [37], [38], [39]. It has been shown that STN-DBS can affect the neural activity of these structures, as well as increase dopaminergic transmission in the striatum [40], [41], [42]. Moreover, the medial part of the STN is adjacent to the medial forebrain bundle which contains essential projections underlying reward functions [15]. Extensive research has demonstrated a close relationship between the mesolimbic system, medial forebrain bundle and ventral pallidum in motivational desire for food rewards, increase in food intake and obesity [43], [44], [45], [46]. Therefore, it seems plausible that an active electrode in the proximity of the medial STN could be ideally positioned to stimulate the reward system, thereby contributing to changes in motivational behaviors related to food intake and weight gain. Our previous study supports this hypothesis, as it revealed that postoperative weight gain correlated with arousal ratings from food pictures in the STN-DBS ON condition, suggesting an altered attribution of incentive salience (i.e., emotional relevance) to rewarding stimuli [47].

Although most of the subjects did not report any changes in food intake, hunger or appetite in our study, the inaccuracy of self-reported intake [48], [49], [50] should prompt caution in the interpretation of these results. Food intake depends largely on reward or homeostatic systems and is only partly under cognitive control [45], [51], [52]. We can hypothesize that slight individual changes in motivational behavior and reward system induced by DBS of subcortical structures need not be reflected in subjective feelings such as hunger or appetite [47], [53]. Further prospective studies taking into account changes in sensitivity to reward [45] and actual food intake would be necessary to clarify this question.

In agreement with another study [7], we found a significant inverse correlation between preoperative body weight and postoperative weight gain. Since weight has been reported to decrease with PD progression [54], it has been suggested that patients treated with DBS normalize their weight compared to their premorbid status because of motor improvement [4], [5]. However, this hypothesis cannot fully account for the fact that although most patients indicated for DBS are normal weight or overweight, the majority of them experience continuous weight gain after surgery [2], [7]. Yet it seems that changes in motor manifestations and energy expenditure can only partly explain both the weight loss in PD and weight gain after initiation of DBS [30], [54], [55]. It has been shown that overweight and obese individuals have higher sensitivity to reward which predicts the tendency for overeating and strengthens preferences for sweet and fatty foods [45]. We speculate that if STN-DBS increases sensitivity to reward in relation to the medial contact site in the subthalamic area, thereby modulating eating behavior, this effect would be more pronounced especially in patients with preoperatively lower body weight, lower sensitivity to reward and without previous, excessive caloric intake.

Some limitations have to be taken into account when interpreting our results. Since body weight may be reflected in local white matter changes [29] and the size and position of the STN varies [56], [57] to some extent relative to the midcommisural point, the influence of anatomic variability cannot be excluded from our measurements. However, we compensated for the variable width of the third ventricle, which significantly affects the mediolateral position of the STN [57], [58], by measuring the x-coordinate from the wall of the third ventricle.

In conclusion, our findings support the hypothesis that weight gain in PD patients treated by STN-DBS may, at least in part, result from the regional effect of stimulation on adjacent structures involved in the central regulation of energy balance or reward.
